# A Patient Registry for the Management of Uterine Fibroids in Canada: Protocol for a Multicenter, Prospective, Noninterventional Study

**DOI:** 10.2196/10926

**Published:** 2018-11-20

**Authors:** Mohamed A Bedaiwy, Peter Janiszewski, Sukhbir S Singh, CAPTURE Steering Committee

**Affiliations:** 1 Department of Obstetrics and Gynecology The University of British Columbia Vancouver, BC Canada; 2 Department of Medical Affairs Allergan Inc Markham, ON Canada; 3 Department of Obstetrics, Gynecology and Newborn Care Ottawa Hospital Research Institute The University of Ottawa Ottawa, ON Canada

**Keywords:** Canada, leiomyoma, registries

## Abstract

**Background:**

Uterine fibroids are the most common benign tumor in women. Among those with fibroids, approximately 30% become symptomatic, with abnormal uterine bleeding, pelvic pain, and bulk symptoms. Despite the high prevalence of fibroids, little information is available regarding symptoms, treatment choices, and outcomes for patients.

**Objective:**

A Canada-wide patient registry was established to understand the real-world practice. This registry included patient presentation and treatment preferences, health care provider attitudes, and clinical outcomes in the management of symptomatic uterine fibroids.

**Methods:**

This study is a prospective, noninterventional, observational patient registry. It will include women diagnosed with uterine fibroids and being managed for symptoms. Participant inclusion criteria were (1) at least 18 years of age, (2) premenopausal with a confirmed diagnosis of uterine fibroids, and associated symptoms, and (3) initiating treatment (drug intervention, procedure intervention, or a combination of both) or watchful waiting. Patients (or legal representative) must understand the nature of the project and provide written informed consent before enrollment. Participant exclusion criteria were (1) they have known or suspected clinically significant pelvic pathology not associated with uterine fibroids, and (2) they are undergoing an emergency hysterectomy at the initial visit. Outcomes will be evaluated in the context of routine clinical practice.

**Results:**

Participant recruitment of this registry began in July 2015. This study currently has a total sample of 1500 patients.

**Conclusions:**

This registry, a first in Canada, will accumulate evidence on the risks and benefits of watchful waiting, and medical and procedural interventions. It will contribute to enhancing access to treatment options for patients.

**Trial Registration:**

ClinicalTrials.gov NCT02580578; https://clinicaltrials.gov/ct2/show/NCT02580578 (Archived by WebCite at http://www.webcitation.org/6yax4Hpvr)

**International Registered Report Identifier (IRRID):**

RR1-10.2196/10926

## Introduction

Uterine fibroids (UF) are benign, monoclonal, hormone-sensitive, smooth muscle tumors that represent the most common solid pelvic tumor in women [1-3]. They affect 20% to 40% of women during their reproductive years [4]. Despite their prevalence, very little is known about the etiology of the disease, burden of illness, and quality of life associated with the condition. Although most patients with UF are asymptomatic, UF can cause heavy and prolonged menstrual bleeding, pelvic pressure or pain, bulk symptoms, and reproductive dysfunction. Patients with UF show increased use of analgesics, sanitary products, and health care resources. There are more physician visits, increased absenteeism, and a decline in energy, and overall activities, including sexual activity [5]. Apart from epidemiologic data, the natural history of UF is poorly understood.

In addition to the palpation of an enlarged irregular uterine contour on pelvic examination, ultrasonography is used to confirm the diagnosis of UF [6]. Recently magnetic resonance imaging (MRI) has gained popularity, but the cost and access to this technology prohibit routine screening. In a uterus with fewer than 5 lesions, ultrasonography appears to be equally effective as detection by MRI and virtually identical for assessing their size and location [7]. However, when the number of lesions is higher, MRI exceeds the technical limitations of ultrasound in precise fibroid mapping and characterization.

A significant issue in the field of fibroid identification is the necessity for differentiating fibroids from other pathologies such as adenomyomas and uterine neoplasms. Although uncommon in women less than 40 years old [8], these conditions require the differential diagnosis with fibroids because the treatment options and prognosis may differ [9]. Patterns of use and accuracy of diagnostic imaging of UF have not been studied systematically in the publicly funded Canadian health care system.

Treatment options for UF patients should ideally address 3 goals: (1) relief of signs and symptoms, (2) sustained reduction in fibroid size, and (3) retention or enhancement of fertility if desired. At the same time, treatment should also aim to improve quality of life, minimize side effects, be convenient for patients, and target UF directly [10]. Medical, procedural, or combined treatment options are available for patients with symptomatic UF. However, there were no medications to treat these women in Canada until 2013 when ulipristal acetate (UPA) was approved.

UPA has shown to be more effective than placebo [11] and as effective as the injectable gonadotropin-releasing hormone agonist leuprolide acetate [12] for treating UF symptoms. Leuprolide acetate received Health Canada indication for preoperative improvement of anemia in symptomatic UF women in 2017 [13]. The uptake, long-term clinical and patient-reported outcomes, efficacy, safety, as well as the pharmacoeconomics of indicated and off-label treatment options for these patients in Canada, have yet to be determined.

In the past 15 years, there has been only 1 completed registry for patients with UF who have undergone uterine artery embolization [14]. In the United States, the COMPARE-UF trial is underway [15]. A Canadian prospective patient registry provides the opportunity to address many unanswered questions, particularly the management patterns and outcomes associated with different treatments for UF and patients’ preferences for, and satisfaction with, managing UF symptoms.

With this background in mind, the objectives of this study are to (1) enhance the understanding of the natural history of symptomatic UF, (2) understand Canadian provider and patient preferences for managing symptomatic UF, (3) examine factors that have an impact on the uptake of UF treatment options and compliance with therapy, (4) examine the effectiveness and efficiency of UF treatment options across ethnicities in real-world practice in Canada, and (5) provide real-world data characterizing long-term clinical and patient-reported outcomes, including effectiveness and safety, associated with different UF management options.

## Methods

### Overall Study Design and Plan

The CAnadian women wiTh Uterine fibroids REgistry (CAPTURE) is a prospective, observational cohort study (NCT02580578) that will include women who have been diagnosed with symptomatic UF. Outcomes will be evaluated as part of routine clinical practice. The target study population will include patients with a diagnosis of UF from 20 study sites across Canada. Participating physicians will not be required to perform any medical procedure that is outside their normal clinical practice. Serious and nonserious adverse drug reactions (ADRs) will be followed up as per local standard of care and as expected in an observational research study. Similarly, both serious and nonserious adverse events (AEs) during and following procedural intervention will be reported. Each study site was responsible for local ethics review board approval as per their institutional policy.

### Participants

Approximately 1000 patients will be enrolled from 20 Canadian investigational sites, including both academic and community practices. Inclusion criteria for the study are premenopausal women at least 18 years of age with confirmed diagnosis and symptoms associated with UF that are being observed (watchful waiting), currently being treated, or initiating treatment (drug intervention, procedure intervention, or a combination of both). All patients (and the patient’s authorized legal representative) should understand the nature of the project and provide written informed consent before or at the initial study visit. Exclusion criteria for the study are known or suspected significant pelvic pathology not associated with UF and patients undergoing an emergency hysterectomy at the initial visit.

### Withdrawal of Participants

Participation in this registry is voluntary, and patients may withdraw at any time and for any reason. Once the patient informs the site staff that she wishes to withdraw her participation, a “Withdrawal from registry” form will be completed with the date and reason. The patient will be informed that all data collected up to that point will be used in the analysis. From that point on, the patient will no longer be contacted for the registry, and no further data on that participant will be collected.

### Participants Lost to Follow Up

Efforts will be made to keep patients in the registry for at least 24 months. A patient will be considered to have missed a study visit after all efforts have been made by site personnel to contact the patient to reschedule within 1 month of the missed appointment. If a patient has missed a follow-up visit, all efforts should be made to reach the patient to ensure that subsequent follow-up visits are completed.

### Study Visits

The visit schedule is comprised of 4 study visits (baseline, 3- to 6-month, 12-month, and 24-month; Table 1) and pre-, intra-, and postoperative visits for patients undergoing procedural interventions (Table 2).

### Baseline Visit

The baseline visit will involve (1) obtaining informed consent, (2) checking for inclusion/exclusion criteria, (3) collecting demographic information, (4) obtaining a medical history, and (5) assessing patient current health status. This will be followed by patient assessments that involve questions on UFs using the EuroQol 5-Dimensions 5-Levels (EQ-5D-5L) survey, the Uterine Fibroid Symptom and Quality of Life (UFS-QOL) questionnaire, the Aberdeen Menorrhagia Severity Scale (AMSS) questionnaire, and evaluation for concomitant medications and history of previous procedural interventions.

The EQ-5D-5L [16] is a validated 5-question survey with a visual analog scale used to measure health outcomes that provides a simple descriptive profile and a single index value for health status. The UFS-QOL [17] is a validated 37-question disease-specific symptom and health-related quality of life questionnaire for UF. It has been used extensively in studies to assess the impact of the disease in patients and treatment options in randomized clinical trials. The AMSS questionnaire is a validated 15-question survey that evaluates the severity and effect of bleeding that occurs during and between menses. There are 7 baseline questions for UF patients asking about the purpose of their visit and what outcome they are hoping to achieve.

If deemed eligible for registry participation, the patient will return for a visit in 3 to 6 months. When available, imaging and other test results (biopsies, blood work) will also be documented.

**Table 1:** Schedule of events of the CAPTURE study at baseline and scheduled visits.

Examinations	Scheduled follow-up visits			
	Baseline	3- to 6-month^a^	12-month^a^	24-month^a^
Signed informed consent form^b^	✓	—	—	—
Inclusion/exclusion criteria	✓	—	—	—
Demographics	✓	—	—	—
Medical history	✓	—	—	—
Fertility wishes	✓	✓	✓	✓
Blood work (hemoglobin)^c^	✓	✓	✓	✓
Gynecologic symptoms	✓	✓	✓	✓
Ultrasound of fibroids	✓	✓	✓	✓
Type and location of fibroids	✓	—	—	—
Endometrial biopsy^c^	✓	—	—	—
Planned/updated management plan	✓	✓	✓	✓
UFS-QOL^d^	✓	✓	✓	✓
Bleeding assessment^e^	✓	✓	✓	✓
EQ-5D-5L^f^	✓	✓	✓	✓
Concomitant medications	✓	✓	✓	✓
Adverse events	—	✓	✓	✓

^a^Visits can be performed by telephone or in clinic; however, an in-person visit is preferred.

^b^Must be obtained from patient prior to study enrollment.

^c^To be captured if available.

^d^UFS-QOL: Uterine Fibroid Symptom and Quality of Life questionnaire.

^e^Tool for assessment is the Aberdeen Menorrhagia Severity Scale questionnaire.

^f^EQ-5D-5L: EuroQol 5-dimensions 5-levels survey used to measure health status.

**Table 2:** Schedule of events of the CAPTURE study at procedural intervention and hysterectomy.

Examinations	Procedural intervention^a^			Hysterectomy
	Preoperative	Intraoperative	Postoperative	1-year follow up
Signed informed consent form	—	—	—	—
Inclusion/exclusion criteria	—	—	—	—
Demographics	—	—	—	—
Medical history	—	—	—	—
Fertility wishes	—	—	—	—
Blood work (hemoglobin)^b^	✓	—	—	—
Gynecologic symptoms	—	—	—	✓
Ultrasound of fibroids	✓	—	—	—
Type and location of fibroids	—	—	—	—
Endometrial biopsy	—	—	✓	—
Planned/updated management plan	✓	—	—	—
UFS-QOL^c^	✓	—	✓	✓
Bleeding assessment^d^	✓	—	✓	—
EQ-5D-5L^e^	✓	—	✓	✓
Concomitant medications	✓	—	—	—
Adverse event	✓	✓	✓	—

^a^Procedural intervention is not mandatory in the registry, and data will be collected if physician and patient decide on a procedural intervention to address symptoms associated with uterine fibroids.

^b^To be captured if available.

^c^UFS-QOL: Uterine Fibroid Symptom and Quality of Life questionnaire.

^d^Not required if patient undergoes hysterectomy procedure. Otherwise the tool for assessment is the Aberdeen Menorrhagia Severity Scale questionnaire.

^e^EQ-5D-5L: EuroQol 5-dimensions 5-levels survey used to measure health status.

### Visits at Three to Six Months, Twelve Months, and Twenty-Four Months

These visits can be performed in the clinic or via telephone; however, an in-person visit is preferred. Participants will be evaluated for updates and an assessment of the management plan, and for ADRs (if medication was prescribed at the previous visit). Also, patient questionnaires (EQ-5D-5L, UFS-QOL, and AMSS) will be administered. Follow up will occur after the baseline meeting at 3 to 6 months, 12 months, and 24 months.

### Preoperative Visit

This visit will only occur if the patient and physician agree on a procedural intervention. During this visit, participants will be evaluated for updates and an assessment of the management plan, and for ADRs (if medication was prescribed at the previous visit). Also, patient questionnaires (EQ-5D-5L, UFS-QOL, and AMSS) will be administered.

### Intraoperative Visit

This visit will only occur if the patient and physician agree on a procedural intervention. During this visit, information on the procedure performed and the AEs will be obtained. Specific details on the type of procedure, route, and surgical times will be recorded. Intraoperative description of outcomes, including morcellation (if performed), will be documented. In the case of myomectomy, fibroid cleavage planes and density (subjective) of the fibroids will be obtained as well.

### Postoperative Visit

This visit will only occur if the patient and physician agree on a procedural intervention. During this visit, information on AEs following the procedure, pathology, and patient questionnaires (EQ-5D-5L, UFS-QOL, and AMSS) will be recorded.

### Hysterectomy Postoperative Follow Up

If a patient undergoes a hysterectomy, a follow-up visit will occur 1 year after the hysterectomy (in addition to the postoperative visit). This follow up will replace all remaining scheduled follow-up study visits. Information on postoperative symptoms, overall satisfaction, UFS-QOL-Hysterectomy, and EQ-5D-5L will be obtained. Once this follow up is completed, the patient will be deemed to have completed all study visits.

### Unscheduled Visits

Unscheduled visits (other than the prescribed appointments) are any unexpected visits, including emergency assessments or changes to care plans. Participants will be assessed for the reason of the visit, update and evaluation of the management plan, and ADRs (if medication was previously prescribed).

### Pregnancy Follow Up

If an unplanned pregnancy occurs with hormonal medical therapy during this study, a serious AE will be recorded. Also, the “Pregnancy follow up” form will be completed to capture baseline pregnancy information. If the patient consents to be followed up on the outcome of the pregnancy, then postdelivery follow-up information will be collected once available. No study visits will be completed during the pregnancy. Following delivery, the patient will resume the study visit schedule until completion.

### Assessment of Clinical Efficacy and Safety

This is a patient registry that tracks the routine practice and management of patients with UF in Canada. The physician will collect data at the initial and follow-up visits between 3 to 6 months, 12 months, and 24 months. Also, if a procedural intervention is performed, pre-, intra-, and postoperative data will be captured. Patients will be asked to complete the EQ-5D-5L, UFS-QOL, and the brief bleeding questionnaires as per the visit schedule outlined in Tables 1 and 2.

### Duration of Study

The duration of the study will be 2 years from the recruitment of the final patient, which occurred in February 2018. It is expected that the last visit of the final patient will occur in February 2020.

### Statistical Methods

The analysis will include all patients enrolled in the study who have been entered into the database.

### Statistical Analysis

The goal of this study is to establish a registry of Canadian patients with symptomatic UF. We have recruited a sample of 1500 patients to help describe the management of UF in this population.

Initially, exploratory data analysis will summarize all variables of interest by reporting means and standard deviations for continuous variables, and frequencies (percentages) for categorical variables. Analysis of variance will be used to compare continuous variables between treatment groups of interest. Chi-square tests will be used to compare categorical variables between the 2 groups. A linear mixed model will be employed to examine the association between treatment groups while adjusting for other covariates of clinical interest. These variables include demographics, UF history, comorbidities, medications, and medical tests (both current and previous).

The model will include a time-by-treatment-interaction term to determine whether treatments have improved quality of life relative to other treatment groups. Furthermore, a random effect will be included at the patient level to incorporate correlation arising from the repeated measures design of the study.

Since this is an observational registry, missing data and drop-outs (ie, early study termination) are expected. Statistical techniques will be used to examine the missing data (including logistic regression) and, if feasible, multiple imputations will be used to make any adjustments.

Additionally, where imputation techniques are not adequate, sensitivity analysis will be employed. All parameter estimates, confidence intervals, and *P* values will be reported from the model, and a 2-sided significance level of .05 will be used.

The original terms used by participating physicians to identify ADRs will be coded using the Medical Dictionary for Regulatory Activities (MedDRA). The MedDRA system organ class and preferred term will be used to present the number and percentage of patients with ADRs, the drug relationship, seriousness, and intensity of ADRs. Chi-square tests will be employed to compare the number of ADRs between groups of interest.

### Data Management Process

Overall coordination of the registry will be led by a steering committee that includes 6 academic gynecologists and 1 community gynecologist. Committee responsibilities include establishing the registry database and protocol, appointing a scientific committee for data analysis and interpretation, and governing the dissemination of the registry findings through a publication plan. All members of the steering committee have a vote on decision making, with the Chair providing a deciding vote in the event of a tie. The study sponsor, Allergan Canada, does not have a vote or seat on the steering committee but will facilitate meetings.

All study data will be captured on HealthDiary, which is a Web-based electronic data-capture system. Data will be managed by the Applied Health Research Center (AHRC), a service unit within the HUB Health Research Solutions and fully integrated with the Li Ka Shing Knowledge Institute of St. Michael’s Hospital, Toronto, Ontario, Canada. The AHRC is a full-service clinical research coordinating center staffed with highly trained individuals including statisticians, methodologists, research coordinators, contract and finance managers, and research informatics specialists. The facility will serve as the data management center. It has managed large randomized trials, cohort studies, and patient registries across provincial and international borders, ensuring privacy regulations using state-of-the-art, Web-based, data management software. The center will be responsible for developing the electronic case report form (eCRF), conducting data management, and issuing and clarifying queries with the participating sites. The research database includes automated edit and logic checks to assist with the data management activities. Data entry screens and edit checks will be tested with mock patient data and removed from the database before beginning the study. In addition to programmed edit and logic checks, manual data verification, and consistency checks will be performed to ensure valid, relevant, and complete data for analysis.

Medical history and concomitant medical conditions/diseases will be coded according to MedDRA. Data handling procedures, including edit check specifications, data entry guide, authorized corrections, coding instructions, and locking procedure, will be detailed in the study data management plan.

### Data Quality Assurance

The database will be secured and enable quality control to be performed at the time of data entry. Quality control will focus on value ranges and missing data. Any discrepancies that occur during eCRF data entry will be flagged in real time by the system and will be saved until addressed by the data entry personnel.

In compliance with Good Clinical Practice and regulatory requirements, the sponsor, a third party on behalf of the sponsor, health regulatory agencies, or research ethics boards may conduct quality assurance audits at any time during or following the study. The participating investigator must agree to allow auditors direct access to all project-related documents, including source documents, and must agree to allocate their time and the time of their project staff to the auditors to discuss findings and issues.

## Results

Participant recruitment for this registry began in July 2015. The registry has recruited 1500 patients, with the first patient enrolled in the second half of 2015. Patient follow up will occur for up to 2 years, with the last patient visit occurring in 2020. It is anticipated that national/industrial funding will be available to continue this initiative beyond 2020 based on the information generated from this registry.

## Discussion

### Importance of the CAPTURE Registry

Despite the prominence of UF and their detrimental effects on patient health, little is known about many aspects of the condition, including the etiology of the disease, the natural history of UF, the impact of UF on fertility and pregnancy outcomes, and the effectiveness of UF treatments in real-world clinical practice. There are virtually no well-designed, long-term, epidemiologic studies published in this disease area. The evidence on the comparative long-term effectiveness of treatment options is also of poor quality. The Agency for Healthcare Research and Quality (AHRQ) has published reviews on the state of the evidence for the management of UF in 2001 and 2007. A more recent review was published by Singh and Belland [10] outlining clinical data on the medical and surgical management of UF along with a treatment algorithm to assist health care professionals.

Our literature search for established UF registries revealed only 1 in the past 15 years called the FIBROID Registry. This was a multicenter, prospective, longitudinal study of the short- and long-term outcomes of uterine artery embolization [14]. Despite the lack of data on the management of UF, the future of obtaining real-world data in this area appears promising. In addition to this registry in Canada, registries in both the United States and Europe have been announced or are currently underway. In the second half of 2014, the Patient-Centered Outcomes Research Institute and AHRQ announced that the Duke University School of Medicine’s Department of Obstetrics and Gynecology and the Duke Clinical Research Institute will lead a 5-year patient registry to evaluate the effectiveness of different treatment strategies for UF patients.

In Canada, there is a lack of documented data characterizing the UF patient population, treatment options, and outcomes associated with women who have UF. Also, no data exist regarding patient preference and satisfaction related to their management. Moreover, with the introduction of UPA—the first Health Canada-approved medication for the treatment of UF—an evaluation of effectiveness and safety is desirable. It should be noted that UPA is undergoing a clinical investigation in the United States and is currently not approved. In Europe, a prospective, observational, noninterventional study is ongoing to document real-world data related to current treatment patterns and outcomes associated with UPA in patients with symptoms associated with UF. This registry is collecting valuable real-world data in Canada on the medical and procedural management of UF to address these issues.

### Potential Limitations

This registry has some limitations. The instruments used at different visits are self-reported, which may result in reporting bias. The noninterventional design can lead to selection bias as well as confounding. Finally, failure to follow up and the expected attrition of the participants at subsequent visits following recruitment could impact on the sample size, thus limiting the external validity.

## Abbreviations

ADR: adverse drug reaction
AE: adverse event
AHRC: Applied Health Research Center
AHRQ: Agency for Healthcare Research and Quality
AMSS: Aberdeen Menorrhagia Severity Scale
CAPTURE: CAnadian women wiTh Uterine fibroids REgistry
eCRF: electronic case report form
EQ-5D-5L: EuroQol 5-dimensions 5-levels
MedDRA: Medical Dictionary for Regulatory Activities
MRI: magnetic resonance imaging
UF: uterine fibroids
UFS-QOL: Uterine Fibroid Symptom and Quality of Life questionnaire
UPA: ulipristal acetate
